# Alfalfa weevils (Coleoptera: Curculionidae) in the western United States are resistant to multiple type II pyrethroid insecticides

**DOI:** 10.1093/jee/toad218

**Published:** 2023-11-25

**Authors:** Erika A Rodbell, Christopher G Caron, Silvia I Rondon, M Umer Masood, Kevin W Wanner

**Affiliations:** Department of Plant Science and Plant Pathology, Montana State University, 119 Plant BioScience, Bozeman, MT 59717-3150, USA; Department of Plant Science and Plant Pathology, Montana State University, 119 Plant BioScience, Bozeman, MT 59717-3150, USA; Oregon IPM Center, Oregon State University, Coast Range Building, 4575 Research Way, Corvallis, OR 97333, USA; Department of Plant Science and Plant Pathology, Montana State University, 119 Plant BioScience, Bozeman, MT 59717-3150, USA; Department of Plant Science and Plant Pathology, Montana State University, 119 Plant BioScience, Bozeman, MT 59717-3150, USA

**Keywords:** insecticide resistance, field crop, forage crop, crop protection, Curculionidae

## Abstract

The alfalfa weevil (*Hypera postica* Gyllenhal (Coleoptera: Curculionidae)), a key pest of alfalfa (*Medicago* sativa L. (Fabales: Fabacae)) across the US, has developed resistance to pyrethroids lambda-cyhalothrin and zeta-cypermethrin in at least 6 western US states. Unfortunately, 6 pyrethroid active ingredients represent most commercial insecticides registered for alfalfa weevil control in forage alfalfa systems. Thus, the loss of efficacy of this mode of action group due to multiple resistance represents a significant agricultural challenge because of a limited registered alternative mode of actions. To evaluate the extent and severity of resistance among pyrethroids around the United States, laboratory bioassays using larvae from Arizona, California, Montana, Oregon, Washington, and Wyoming, including both the Egyptian and western strains, were conducted. Results indicated that similar degrees of resistance among type II pyrethroids as determined by both laboratory bioassays and field trials exist. The LC_50_ values of alpha-cypermethrin, beta-cyfluthrin and zeta-cypermethrin produced significant correlations with the LC_50_ values of lambda-cyhalothrin. In contrast, resistance did not include type I pyrethroid, bifenthrin (registered for seed alfalfa production), whose LC_50_ values yielded a slope not significantly different from zero when correlated with lambda-cyhalothrin. Field trials conducted in Arizona, Montana, and Washington corroborated laboratory results, as commercial formulations with type II pyrethroid active ingredients failed to adequately control alfalfa weevils resistant to lambda-cyhalothrin. Integrated resistance management recommendations are discussed.

## Introduction

Insecticides are an important component of integrated pest management (IPM), and insecticide resistance challenges IPM strategies (Pedigo et al. 2021, IRAC 2023). In 2002, the annual economic loss due to insecticide resistance in the United States (US) was estimated at $1 billion (Clark and Yamaguchi 2002). Insecticide resistance poses a risk to forage alfalfa (*Medicago sativa* L. (Fabales: Fabaceae)) production in the United States, with evidence of alfalfa weevil (*Hypera postica* Gyllenhal (Coleoptera: Curculionidae)) resistance to pyrethroid active ingredients (mode of action group [MoA] 3A) in the western United States (Rodbell and Wanner 2021, Rodbell et al. 2022, Wanner et al. 2022).

The alfalfa weevil, an invasive insect, is an economically damaging pest of alfalfa in North America (Peterson et al. 1992, Pellissier et al. 2017, Rankin 2020, Levi-Mourao et al. 2022). Larval feeding causes economic damage through defoliation, reducing forage yield, quality, and value (Onstad and Shoemaker 1984, Hutchins et al. 1990). Alfalfa weevil, native to Eurasia, was detected in the United States on 3 occasions, with each introduction recognized as an individual strain. The first introduction occurred in Salt Lake City, UT in 1904 (western strain), the second in Yuma, AZ in 1939 (Egyptian strain), and the third in Maryland in 1952 (eastern strain) (Titus 1910, Wehrle 1940, Poos and Bissell 1953, Hsiao 1993, Bundy et al. 2005). The Egyptian strain is found in the southwestern United States, the western strain is distributed in central California and across the northwestern United States, and the eastern strain is distributed across the central and eastern regions of the United States (Böttger et al. 2013).

Soon after the alfalfa weevil’s invasion, classical biological control was implemented by the United States Department of Agriculture Agricultural Research Service (USDA-ARS) along with the Animal and Plant Health Inspection Service (APHIS), through the introduction of parasitoid wasps (Bryan et al. 1993). These agencies introduced 8 parasitoid wasp species in the US western region, the most successful being the ichneumonid wasps *Bathyplectes curculionis* (Thomson), *B. anurus* (Thomson), and *B. stenostigma* (Thomson) (Bryan et al. 1993, Pellissier et al. 2017). Cultural IPM tactics are limited to grazing and maintaining a healthy alfalfa stand, while mechanical control tactics are limited to harvesting early to salvage yield (Summers 1998, Pellissier et al. 2017, Pedigo et al. 2021).

In the 1950s, insecticides became the primary method to reduce alfalfa weevil damage, and by 1961 the routine application of organochlorine insecticides led to resistance to heptachlor and dieldrin (Bishop 1964, Pedigo et al. 2021). Subsequently, pyrethroids (MoA 3A) became the predominant insecticide used to control alfalfa weevil populations (DPR-PUR 2023). Pyrethroids are divided into 2 types based on their chemical structure, type I pyrethroids lack an α-cyano segment, while type II pyrethroids contain it (Yu 2015). However, after decades of routine use, alfalfa weevils in the western United States have developed resistance to the type II pyrethroids lambda-cyhalothrin and zeta-cypermethrin (Rodbell et al. 2022).

It is not uncommon for insect populations to develop cross or multiple resistance to active ingredients within the same mode of action (MoA) (Yu 2015). Cross-resistance occurs when a single genetic mechanism causes resistance to multiple active ingredients. Multiple resistance to several insecticides results from multiple genetic mechanisms of resistance (Yu 2015). As the mechanism(s) of resistance have not been identified for the alfalfa weevil, we use the term multiple resistance in this context to simply signify similar patterns of resistance between active ingredients and not to imply the involvement of multiple genetic mechanisms.

Currently, there are limited alternative MoA groups available for alfalfa weevil control in forage alfalfa systems, resulting in an increased reliance on indoxacarb (MoA 22A) in areas where pyrethroid resistance has established (Rodbell et al. 2022, DPR-PUR 2023). Pyrethroids represent 6 of the 13 active ingredients registered for alfalfa weevil management in forage alfalfa systems (Vardiman et al. 2022). A seventh pyrethroid, bifenthrin (type I), is registered in the United States for alfalfa seed production (Hanson 2022). With limited management options, the loss of all pyrethroids due to multiple resistance would challenge the management of forage alfalfa production. Insecticide resistance management (IRM) recommendations for alfalfa weevils is needed to address this agricultural challenge. An important first step in developing an IRM strategy is to determine whether resistance to lambda-cyhalothrin includes multiple resistance to all available pyrethroid active ingredients.

Across 3 distinct alfalfa production areas of the western United States, we aimed to (i) test for resistance to multiple pyrethroid active ingredients using field-collected alfalfa weevil larvae in laboratory contact bioassays; (ii) corroborate laboratory results using commercially formulated pyrethroid insecticides applied at field sites with quantified resistance to lambda-cyhalothrin; and (iii) identify the alfalfa weevil strain(s) present at each field trial location to help interpret potential patterns of resistance to the different active ingredients.

## Materials and Methods

### Concentration Response Bioassays

Alfalfa weevils were collected from 6 western states (e.g., AZ, CA, MT, OR, WA, and WY) representing 19 field locations, from 3 June to 26 June 2021 and 14 February to 21 June 2022. Field sampling was partially focused on fields with informed management history from crop advisor, extension agent, and/or producer reports. Using sweep nets (BioQuip, Rancho Dominguez, CA), larvae were collected and placed into19-liter buckets with fresh alfalfa collected from the same field and covered with no-see-um number 20 mesh (Quest Outfitters, Sarasota, FL). Collected larvae were assayed using glass vials treated with 6 pyrethroid active ingredients. An alternative MoA, indoxacarb (MoA 22A), was also tested when larvae were available.

All insecticides used for laboratory bioassays were technical grade material, that included bifenthrin and permethrin (type I) and alpha-cypermethrin, beta-cyfluthrin, lambda-cyhalothrin, and zeta-cypermethrin (type II). When sufficient larvae were available, indoxacarb (MoA 22A) was included in bioassays. Bifenthrin (2-methyl-3-phenylphenyl)methyl (1*R*,3*R*)-3-[(*Z*)-2-chloro-3,3,3-trifluoroprop-1-enyl]-2,2-dimethylcyclopropane-1-carboxylate), batch number BCBX0667, 98% purity; permethrin (3-phenoxybenzyl (1RS,3RS;1RS,3SR)-3-(2,2-dichlorovinyl)-2,2-dimethyl-cyclopropanecarboxylate), batch number 45614, 92% purity; alpha-cypermethrin ([cyano-(3-phenoxyphenyl)methyl] 3-(2,2-dichloroethenyl)-2,2-dimethylcyclopropane-1-carboxylate), batch number BCBT3763, 98% purity; and beta-cyfluthrin ([(*R*)-cyano-(4-fluoro-3-phenoxyphenyl)methyl] (1*S*)-3-(2,2-dichloroethenyl)-2,2-dimethylcyclopropane-1-carboxylate), batch number BCBX0283, 98% purity were purchased from Sigma-Aldrich (St. Louis, MO). Lambda-cyhalothrin (alpha-cyano-3-phenoxybenzyl (Z)-(1RS)-*cis* 3-(2-chloro-3,3,3-trifluoropropenyl)-2,2-dimethylcyclopropanecarboxylate) was provided by Syngenta (Basel, Switzerland), batch number 1071218, 89% purity. Zeta-cypermethrin ([Cyano-(3-phenoxyphenyl)methyl]3-(2,2-dichloroethenyl)-2,2-dimethylcyclopropane-1-carboxylate), batch number 122279866, 92% purity; and indoxacarb, (methyl (4*aS*)-7-chloro-2-[methoxycarbonyl-[4-(trifluoromethoxy)phenyl]carbamoyl]-3,5-dihydroindeno[1,2-e][1,3,4]oxadiazine-4*a*-carboxylate), 98% purity, were provided by FMC Corporation (Newark, NJ).

Detailed methods are outlined in Rodbell and Wanner (2021), Rodbell et al. (2022), and Supplementary Methods S1. Stock solutions and 7 serial dilutions of each of the active ingredient were prepared in 95% acetone. Glass vials (Discount Vials, Madison, WI) were treated with 1 ml of each serial dilution to produce concentrations of 0.0033–10.0 µg/cm^2^ for type II pyrethroids, 0.033–100.0 µg/cm^2^ for type I pyrethroids, and 0.0001–0.1 µg/cm^2^ for indoxacarb. Each bioassay typically included 7 concentrations and a 95% acetone control (*n* = 5 replicated vials, 10 larvae per vial). After 24-h in the dark at room temperature, the larvae were scored as dead or alive after exposure on a heated hot plate (43–50 °C) (Rodbell and Wanner 2021, Rodbell et al. 2022). Probit analysis was used to quantify the lethal concentration of an active ingredient that generated 50% mortality (LC_50_) using Probit Or LOgit analysis (POLO) software (LeOra Software, Parma, MO) as described in Rodbell and Wanner (2021), Rodbell et al. (2022), and Supplementary Methods S1, and Robertson et al. (2017).

### Assessing Multiple Resistance Between Active Ingredients

To assess multiple resistance between pyrethroid active ingredients, larvae from the same field (19 field sites total) were assayed with multiple active ingredients. Seven field sites in AZ, CA, MT, and WA were assayed with bifenthrin, permethrin, alpha-cypermethrin, beta-cyfluthrin, lambda-cyhalothrin, and zeta-cypermethrin. Indoxacarb (MoA 22A) was included as an alternate mode of action comparison with the exception of 2 field sites in MT. The remaining 12 field sites were assayed using at least 3 pyrethroid active ingredients. Resistance ratios were calculated for lambda-cyhalothrin using the average LC_50_ value of the 9 most susceptible western region locations identified by Rodbell et al. (2022), a denominator of 0.013 µg/cm^2^.

Multiple resistance between lambda-cyhalothrin and each pyrethroid active ingredient was tested by correlating their LC_50_ values. A Pearson Product Moment Correlation two-tailed test, conducted with GraphPad Prism Statistical Software (San Diego, CA), was used to determine statistical significance. A positive slope significantly different from zero (*P* ≤ 0.05) supports mutual resistance and the R^2^ value indicates strength of the best-fit line (Smirle et al. 2002, Vassiliou et al. 2010, Min et al. 2014). To further assess multiple resistance with lambda-cyhalothrin, field sites were divided into 2 conservative categories defined by their lambda-cyhalothrin resistance ratios determined in Rodbell et al. (2022). The response of each active ingredient (percentage mortality) to a diagnostic concentration should reflect the lambda-cyhalothrin resistance category if multiple resistance exists (Menger et al. 2020, Rodbell et al. 2022). Fields with resistance ratios <76.9 were considered susceptible or moderately resistant, and fields with resistance ratios >76.9 were considered highly resistant (Rodbell et al. 2022). Bioassay mortality from the acetone control group and the 3.3 µg/cm^2^ active ingredient concentration was analyzed post hoc. Percentage mortality was log-transformed to meet the normality assumptions of ANOVA performed in RStudio statistical software version 1.3.1093 (RStudio Team, Boston, MA) and the Tukey HSD test (*P* = 0.05) was used to identify significant differences among treatments.

### Field Trials

In 2022, 3 field trials were conducted, 1 each in Arizona (La Paz County), Montana (Big Horn County), and Washington (Yakima County). Treatments consisted of 6 commercial formulations applied at their maximum label rates and an untreated check (randomized complete block design, *n* = 5): (i) Beta-cyfluthrin (Baythroid XL, Bayer, Leverkusen Germany), 0.20 liter/ha (2.80 oz/ac); (ii) Lambda-cyhalothrin (Warrior II, Syngenta), 0.14 liter/ha (1.92 oz/ac); (iii) Zeta-cypermethrin (Mustang-Maxx, FMC), 0.29 liter/ha (4.0 oz/ac); (iv) Bifenthrin (Brigade, FMC Corporation), 0.47 liter/ha (6.40 oz/ac); (v) Permethrin (Permethrin, Loveland Products, Inc., Loveland, CO), 0.58 liter/ha (8.0 oz/ac); (vi) Indoxacarb (Steward EC, FMC), 0.83 liter/ha (11.30 oz/ac); and (vii) Untreated check. Each plot measured 3.05 m by 9.14 m and the nonionic surfactant Preference was used with indoxacarb, 0.25% v/v. All insecticides were applied using a Chapin 63924 4-gallon backpack sprayer with a 24-V Rechargeable Battery (Chapin International Inc. Batavia, NY) calibrated to deliver 167.9 liter/ha (18 gallon/ac) at 172.37kPA (25 psi).

Larvae were sampled from each plot using ten 180° sweeps with a standard 38.1 cm diameter sweep net (BioQuip, Rancho Dominguez, CA), 6 (AZ and WA) or 7 (MT) days after treatment (DAT). Total larval counts were log-transformed, and treatment effects analyzed statistically by ANOVA followed by the Tukey HSD test (*P* = 0.05) (RStudio statistical software version 1.3.1093, RStudio Team).

### Identifying Alfalfa Weevil Strain

Larvae from each of the 3 field trial sites were preserved in 95% ethanol and stored at −20 °C. Six individual larvae were arbitrarily selected, rinsed individually with 100% ethanol, and the genomic DNA extracted from each using a Qiagen DNeasy kit (Qiagen, Germantown, MD). Forward and reverse polymerase chain reaction (PCR) primers C1-J-2797 and C2-N-3686 were used to amplify a 927 bp product of the mitochondrial COI/COII gene that was subsequently digested using the AluI restriction enzyme (Qiagen, Germantown, MD) (Erney et al. 1996, Kuwata et al. 2005). After electrophoresis on a 1.2% agarose gel, the western strain was distinguished from the Egyptian/Eastern strains based on the number and size of the resulting restriction enzyme products: 486 and 357 base-pair (bp) products for the Egyptian/eastern strains and 357, 284, 202 bp products for the western strain (Erney et al. 1996, Bundy et al. 2005, Kuwata et al. 2005). To distinguish between eastern and Egyptian strain individuals, PCR primers CB-J-11545 and N1-N-11841 were used to amplify and sequence a 300 bp region of the mitochondrial Cytochrome b/ ND1 genes (Erney et al. 1996). This segment of mitochondrial DNA contains 2 nucleotides that differ by strain (eastern = AC; Egyptian = TT; and western = TC) (Erney et al. 1996). The 300 bp Cytochrome b/ND1 gene PCR product was sequenced in both directions (McLab, San Francisco, CA).

## Results

**Fig. 1.**
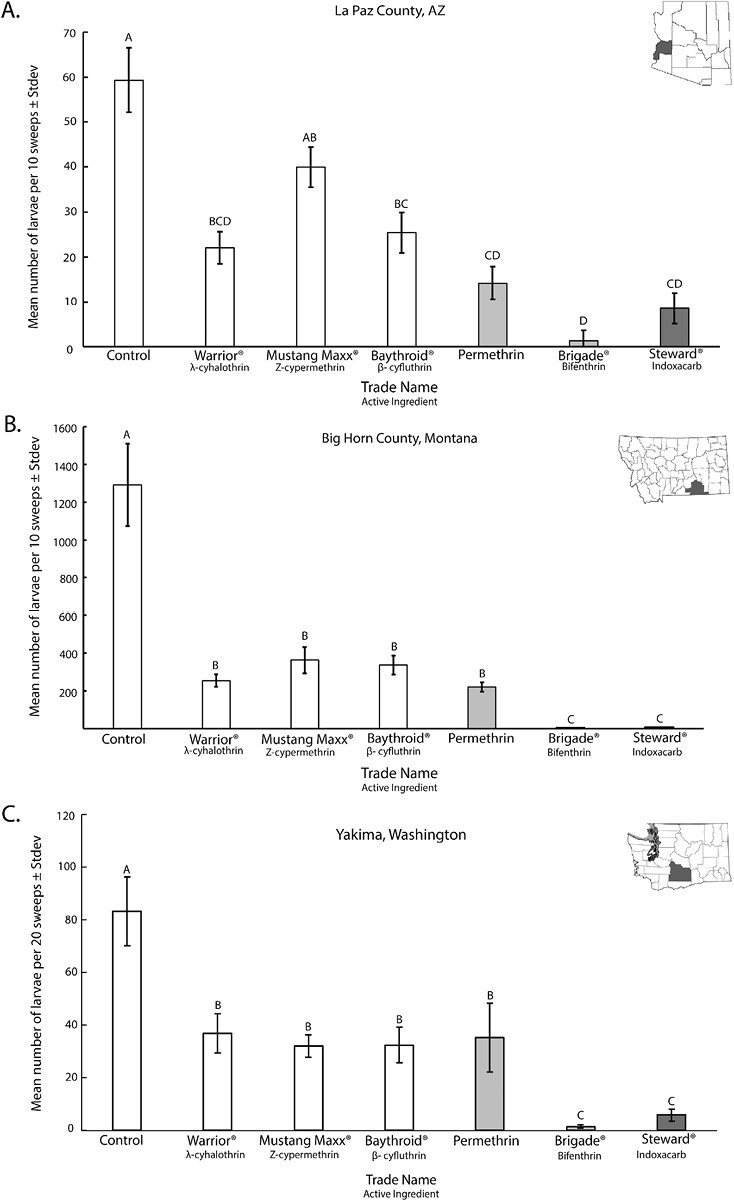
Mean number of alfalfa weevil larvae ($\bar{x\;}$± SD) per 10 sweeps (B & C) or 20-sweeps (A) collected 6–7 days after insecticide application. Field plots were located at Arizona La Paz County Site 1, Montana Big Horn County Site 1, and Washington Yakima County Site 1, with all insecticides applied at their maximum label rate. All sites were classified as highly resistant to lambda-cyhalothrin (Table 2). Type II pyrethroids: Warrior II, Mustang-Maxx, and Baythroid XL. Type I pyrethroids: Permethrin and Brigade. Steward EC was included as alternative MoA group 22A. Treatment effects are significantly different, ANOVA, *F*_6, 28 _= 13.32, *P* < 0.001, Arizona; *F*_6, 28_ = 25.48; *P *< 0.001, Montana; and *F*_6, 28 _= 10.56, *P *< 0.001, Washington. Columns with different letters are significantly different, *P *< 0.05, Tukey pairwise comparison.

**Fig. 2.**
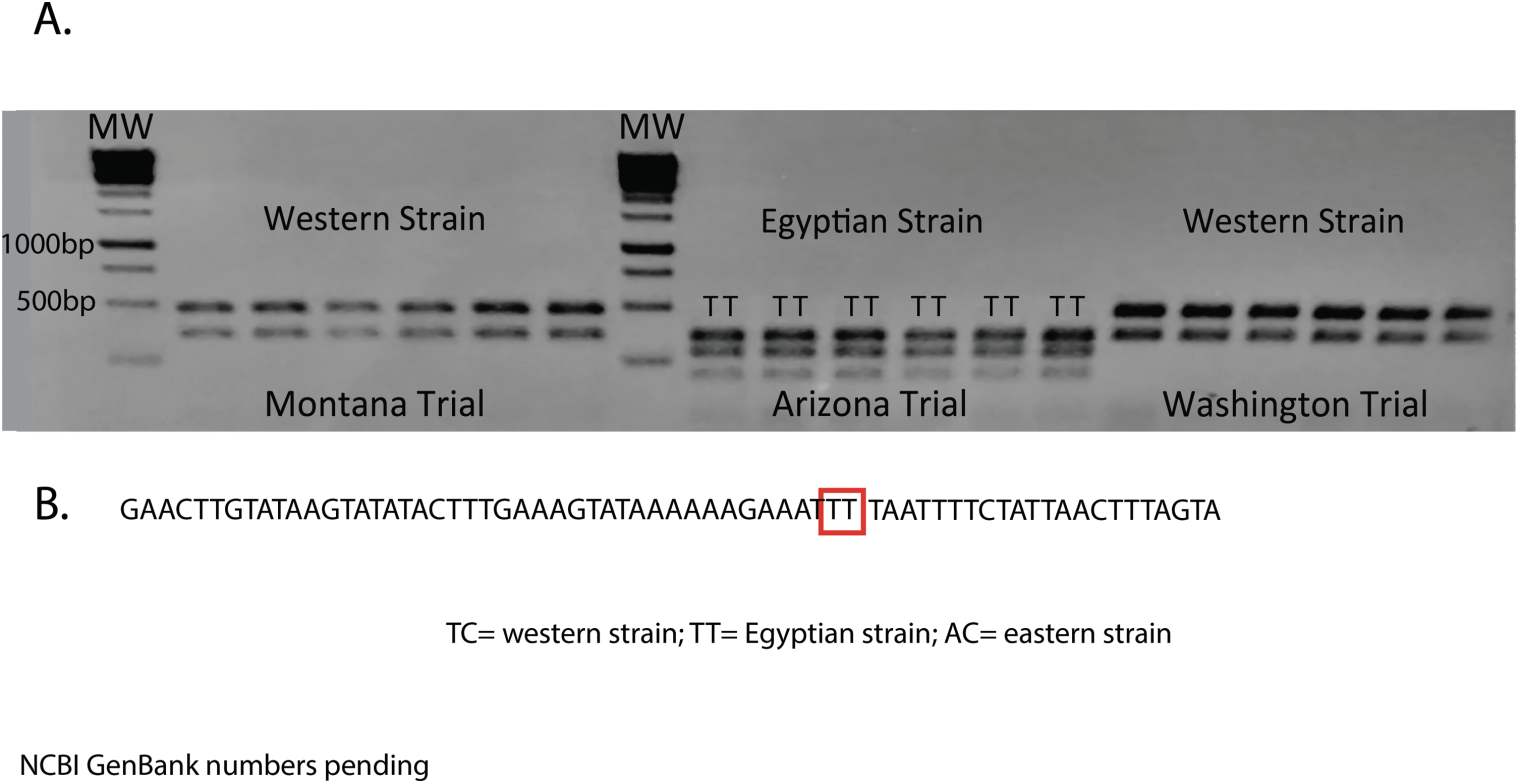
A) Alfalfa weevil strain identification by PCR-Restriction Fragment Length Polymorphism (RFLP). Each lane represents the analysis of a single larva sampled from the field trial sites in Fig. 1B. Partial sequence of the cytochrome b/ND1 mitochondrial gene containing the 2 nucleotide polymorphisms that distinguish Egyptian and eastern strain alfalfa weevils: TT, Egyptian strain; AC, eastern strains. Molecular weight marker (MW) of 1Kb. All 6 larva from the AZ site contained the “TT” polymorphism.

At all field site locations classified as resistant to lambda-cyhalothrin (1.32 to >10 µg/cm^2^), the LC_50_ values for all tested type II pyrethroids were also high: alpha-cypermethrin, 3.38 to >10 µg/cm^2^; beta-cyfluthrin, 1.70 to >10 µg/cm^2^; and zeta-cypermethrin, 3.75–7.75 µg/cm^2^. In contrast, LC_50_ values of type I bifenthrin ranged from 0.01 to 0.86 µg/cm^2^ at any field site tested. Bioassay results using type I permethrin were more variable depending on the field site, ranging from 0.47 to 2.08 µg/cm^2^ in the lower range and 8.43 to >100 µg/cm^2^ in the higher range. Indoxacarb, included as an alternate MoA comparison, consistently yielded low LC_50_ values (0.001–0.02 µg/cm^2^). The AZ, MT, and WA field trial sites were classified as resistant to lambda-cyhalothrin based on bioassay results (Tables 1 and 2). At these sites, larval mortality after the application of commercial formulations of each pyrethroid active ingredient corresponded to the bioassay results. Type II pyrethroids and permethrin failed to provide adequate control while bifenthrin and indoxacarb were effective (Fig. 1). All 6 larvae analyzed from the MT and WA field sites were identified as western strain, while all 6 from the AZ site were Egyptian (Fig. 2).

**Table 1.** To identify the degree of resistance the lethal concentration causing 50% mortality (LC_50_) value for lambda-cyhalothrin was assigned to 3 conservative categories based on its associated resistance ratio, susceptible, moderate, and high resistance (Rodbell et al. 2022)

Resistance Category	LC_50_ (µg/cm^2^)	Resistance ratio (LC_50_/0.013)	Times (X) higher label rate of 0.34 µg/cm^2^
Susceptible	<0.30	<23.08x	<0.9X
Moderate	0.30–1.0	23.08x–76.9x	0.9X–2.9X
High	>1.0	>76.9x	>2.9X

**Table 2.** Lethal concentration of type II and type I pyrethroids (MoA 3A) and indoxacarb (MoA22A) causing 50% mortality (LC_50_) using location samples collected from the 3 lambda-cyhalothrin resistant field trial sites: La Paz County, Arizona (AZ) Field Site 1, Big Horn Co., Montana (MT) Field Site 1, and Yakima County, Washington (WA) Field Site 1 (Tables 3 and 4). No data, (–)

Active ingredient (µg/cm^2^)							
Field Trial	A-cypermethrin	*β*-cyfluthrin	λ-cyhalothrin	Z-cypermethrin	Bifenthrin	Permethrin	Indoxacarb
AZ	>10.00	>10.00	>10.00	3.75	0.51	16.40	0.01
MT	>10.00	9.57	4.50	7.75	0.30	6.44	0.01
WA	3.38	1.70	1.32	–	0.36	100.00	–

### Susceptibility of Larvae From 6 Western US States to Multiple Pyrethroid Active Ingredients

Lambda-cyhalothrin LC_50_ values ranged from 0.06 µg/cm^2^ to > 10 µg/cm^2^, generating RRs from 4.6 to 769, representing locations susceptible, moderately resistant, and highly resistant to lambda-cyhalothrin (*n* = 22; Table 3). For context, the label rate for lambda-cyhalothrin is 0.22–0.34 µg/cm^2^ (0.02–0.03 lb AI/acre; 22.41–33.63 g AI/ha) (Buntin 2020). Type II pyrethroids: alpha-cypermethrin, beta-cyfluthrin, and zeta-cypermethrin all produced similar ranges of LC_50_ values, 0.35 to > 10.00 (*n* = 14), 0.13 to > 10.00 (*n* = 17) and 0.09 to > 10.00 (*n* = 14) µg/cm^2^, respectively (Table 4). Their label rates are similar to lambda-cyhalothrin, generally equivalent to 0.13–0.28 µg/cm^2^, while type I bifenthrin and permethrin are approximately 4–8 times higher (1.12–2.24 µg/cm^2^, respectively) (Buntin 2020). Bifenthrin LC_50_ values ranged from 0.01 to 0.86 µg/cm^2^ (*n* = 15) and permethrin, from 0.47 to > 100.00 µg/cm^2^ (*n* = 21, Table 4).

**Table 3.** Concentration of lambda-cyhalothrin (MoA 3A, type II pyrethroid) causing 50% mortality (LC_50_) using location samples collected from Arizona (AZ), California (CA), Montana (MT), Oregon (OR), Washington (WA), and/or Wyoming (WY). Resistance ratios (RR) were calculated using a common denominator of 0.013 µg/cm^2^ (Rodbell et al. 2022). Probit analysis statistics include the *t-*ratio of the slope, chi-square (χ^2^), and degrees of freedom (df). Each location sample was assigned to 1 of 3 resistance categories: susceptible (S); moderate (M); and high (H) (Table 1)

Active ingredient	State	County	Field site_date_	LC_50_ µg/cm^2^	T-ratio_slope_	χ^2^_df_	*P-*value	RR	R Level
Lambda-cyhalothrin	AZ	La Paz	1_2022_	>10.00	3.26	5.33_2_	0.07	>769.2	H
		Pinal	1_2022_	0.93	5.47	0.40_2_	0.82	71.5	M
	CA	Riverside	1_2022_	1.82	3.87	3.69_1_	0.54	140.0	M
			1_2022_	9.61	4.87	6.38_4_	0.17	739.2	H
	MT	Big Horn	1_2021_	4.50	6.48	4.63_4_	0.33	346.2	H
			1_2022_	7.40	3.09	10.31_5_	0.07	569.2	H
			1_2022_	>10.00	2.64	6.62_5_	0.25	>769.2	H
			2_2021_	8.40	4.65	6.95_5_	0.22	646.1	H
			2_2022_	>10.00	–	–	–	>769.2	H
			3_2021_	0.32	3.54	3.11_1_	0.08	24.6	M
			3_2021_	0.57	6.76	3.61_5_	0.61	43.8	M
		Broadwater	2_2022_	0.39	8.54	7.04_4_	0.13	30.0	M
			2_2022_	0.74	6.09	10.21_5_	0.07	56.9	M
			3_2021_	0.43	5.27	3.45_2_	0.18	33.1	M
		Madison	1_2021_	0.06	7.14	5.03_4_	0.29	4.6	S
			2_2021_	0.26	7.01	3.67_3_	0.30	20.0	S
	OR	Umatilla	1_2022_	0.20	5.84	8.16_4_	0.09	15.4	S
			1_2022_	0.59	4.34	2.38_2_	0.31	45.4	M
	WA	Klickitat	1_2022_	2.00	3.50	8.27_5_	0.14	153.8	H
			2_2022_	3.77	2.52	2.82_2_	0.24	290.0	H
		Yakima	1_2022_	1.32	5.82	3.26_4_	0.51	101.5	H
	WY	Sheridan	1_2022_	>10.00	–	–	–	>769.2	H

**Table 4.** Lethal concentration of type II and type I pyrethroids (MoA 3A) and indoxacarb (MoA 22A) causing 50% mortality (LC_50_) using location samples collected from Arizona (AZ), California (CA), Montana (MT), Oregon (OR), Washington (WA), and/or Wyoming (WY). Probit analysis statistics include the *t-*ratio of the slope, chi-square (χ^2^), and the degrees of freedom (df)

Active Ingredient	State	County	Field site_date_	LC_50_ µg/cm^2^	*T*-ratio_slope_	χ^2^_df_	*P-*value
Alpha-cypermethrin	AZ	Pinal	1_2022_	0.69	4.89	0.22_1_	0.64
	MT	Big Horn	1_2021_	>10.00	3.93	6.51_4_	0.16
			1_2022_	3.23	3.51	0.53_1_	0.47
			2_2022_	2.16	2.05	2.05_1_	0.15
		Broadwater	1_2021_	1.06	3.48	0.65_1_	0.42
			1_2022_	>10.00	2.04	0.43_1_	0.51
			2_2022_	0.35	4.96	0.35_1_	0.55
		Madison	1_2021_	2.47	2.30	0.96_1_	0.33
			1_2022_	1.09	3.55	0.02_1_	0.89
	WA	Klickitat	1_2022_	2.79	4.44	1.57_1_	0.21
	WY	Sheridan	1_2021_	3.37	4.36	9.00_5_	0.11
			1_2022_	2.16	2.05	2.05_1_	0.96
Beta-cyfluthrin	AZ	La Paz	1 _2022_	>10.00	0.65	0.13_1_	0.72
		Pinal	1_2022_	0.32	5.51	0.87_1_	0.35
	CA	Riverside	1_2022_	1.48	3.57	0.20_1_	0.66
	MT	Big Horn	1_2021_	9.57	5.61	0.75_5_	0.98
			1_2022_	4.88	1.96	0.43_1_	0.51
			2_2021_	1.12	4.80	4.50_5_	0.48
			2_2022_	2.29	4.32	0.02_1_	0.88
		Broadwater	1_2022_	0.72	3.54	1.29_1_	0.26
			2_2022_	0.13	4.61	0.06_1_	0.80
			3_2021_	0.87	5.91	0.004_1_	0.95
		Madison	1_2021_	0.43	5.93	0.35_1_	0.55
			2_2021_	0.25	0.05	6.46_1_	0.83
	OR	Umatilla	1_2022_	1.90	4.32	0.50_1_	0.48
	WA	Klickitat	1_2022_	2.14	5.22	2.81_1_	0.09
			2_2022_	8.50	4.00	2.78_1_	0.09
			3_2022_	2.97	1.97	1.15_1_	0.28
		Yakima	1_2022_	1.70	4.60	2.91_1_	0.09
	WY	Sheridan	1_2022_	>10.00	2.21	0.65_1_	0.42
Zeta-cypermethrin	AZ	La Paz	1_2022_	3.75	1.98	1.82_1_	0.18
		Pinal	1_2022_	0.65	5.86	0.50_1_	0.48
Active Ingredient	CA	Riverside	1_2022_	>10.00	2.02	0.15_1_	0.70
	MT	Big Horn	1_2021_	7.75	6.32	10.95_5_	0.052
			1_2022_	4.48	2.27	0.30_1_	0.59
			2_2021_	1.23	6.90	8.75_5_	0.12
			3_2021_	0.53	5.13	0.0002_1_	0.99
		Broadwater	1_2021_	0.09	5.20	1.02_1_	0.31
		Madison	1_2021_	0.32	7.31	1.58_4_	0.81
			2_2021_	0.28	6.52	4.79_4_	0.31
	WA	Klickitat	2_2022_	>10.00	2.23	1.06_1_	0.30
			3_2022_	1.44	2.15	3.20_1_	0.07
	WY	Sheridan	1_2021_	5.82	5.21	10.62_5_	0.06
			1_2022_	8.79	2.67	1.29_1_	0.26
Bifenthrin	AZ	Pinal	1_2022_	0.05	4.66	1.09_5_	0.95
	CA	Riverside	1_2022_	0.27	5.13	1.10_1_	0.29
	MT	Big Horn	1_2022_	0.11	4.94	0.71_1_	0.40
			2_2021_	0.14	3.24	0.47_4_	0.98
			2_2022_	0.15	3.24	0.64_1_	0.42
		Broadwater	1_2021_	0.01	5.00	0.01_1_	0.99
			2_2022_	0.73	5.94	0.32_1_	0.57
		Madison	1_2021_	0.11	3.75	1.59_3_	0.66
			2_2021_	0.01	6.01	0.08_1_	0.77
	OR	Umatilla	1_2022_	0.09	6.10	4.39_2_	0.11
	WA	Klickitat	1_2022_	0.39	7.74	0.76_2_	0.68
			2_2022_	0.86	7.78	1.51_1_	0.22
			3_2022_	0.04	5.41	5.04_2_	0.08
		Yakima	1_2022_	0.36	5.56	1.21_2_	0.54
	WY	Sheridan	1_2022_	0.19	6.70	1.57_2_	0.46
Permethrin	AZ	La Paz	1_2022_	16.37	3.89	2.78_1_	0.10
		Pinal	1_2022_	17.04	4.68	0.03_1_	0.87
	CA	Riverside	1_2022_	16.37	2.78	2.78_1_	0.10
			2_2022_	1.99	3.61	0.01_1_	0.94
	MT	Big Horn	1_2021_	6.44	4.49	5.10_3_	0.16
			1_2022_	19.72	6.04	4.29_2_	0.12
			2_2021_	3.83	5.11	0.62_2_	0.73
			2_2022_	8.43	3.36	6.04_2_	0.05
			3_2021_	2.81	5.59	0.19_1_	0.67
		Broadwater	1_2021_	2.67	6.63	8.92_4_	0.06
			1_2022_	24.18	3.41	3.29_2_	0.19
			3_2021_	0.47	5.80	0.26_1_	0.61
		Madison	1_2021_	1.92	5.57	4.74_4_	0.31
			2_2021_	2.08	7.23	3.12_4_	0.54
	OR	Umatilla	1_2022_	4.62	3.97	0.30_1_	0.58
	WA	Klickitat	1_2022_	9.43	5.83	0.52_1_	0.47
Active Ingredient			2_2022_	12.67	5.17	0.81_2_	0.67
		Yakima	1_2022_	100.00	2.94	0.76_1_	0.76
	WY	Sheridan	1_2021_	3.55	8.55	5.61_4_	0.23
			1_2022_	14.01	5.45	0.93_1_	0.33
Indoxacarb	AZ	La Paz	1_2022_	0.01	3.93	0.27_1_	0.61
		Pinal	1_2022_	0.001	5.22	2.23_1_	0.13
	CA	Riverside	1_2022_	0.01	4.40	0.24_1_	0.62
	MT	Big Horn	1_2022_	0.01	3.15	8.03_5_	0.15
			2_2022_	0.02	6.08	8.00_4_	0.09
	OR	Umatilla	1_2022_	0.01	5.60	6.02_4_	0.20
	WA	Klickitat	1_2022_	0.01	8.64	1.56_5_	0.91
	WY	Sheridan	1_2022_	0.01	6.09	3.56_5_	0.61

### Resistance of Alfalfa Weevil to Multiple Type II Pyrethroid Active Ingredients

**Fig. 3.**
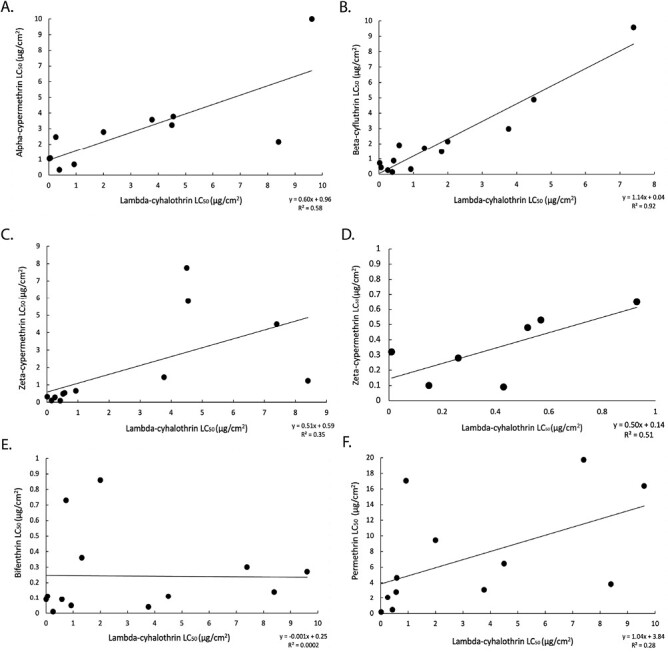
Correlation between pyrethroid active ingredients with the y-axis representing the lethal concentration causing 50% mortality (LC_50_) of a pyrethroid active ingredient, and the X-axis representing lambda-cyhalothrin (type II pyrethroid, MoA 3A) LC_50_ values. Each marker represents a location sample and its associated LC_50_ values. Correlations help determine the presence or absence of multiple resistance between lambda-cyhalothrin and other type II (e.g., alpha-cypermethrin) or type I (e.g., bifenthrin) active ingredients. Multiple resistance can be identified via the R^2^ value and the *P*-value of the slope of the line of best fit. Regarding cross-resistance and multiple resistance, the *P*-value signifies if the slope of the line of best fit is significantly different from zero. In this case, the line of best fit has identified multiple resistance among type II pyrethroids (i.e., alpha-cypermethrin, beta-cyfluthrin, and zeta-cypermethrin, A-D), lack of multiple resistance to the type I pyrethroid bifenthrin (E), and moderate multiple resistance to the type I pyrethroid permethrin (F), with increased resistance to the type II pyrethroid lambda-cyhalothrin. Figure D, illustrates the correlation among location samples encompassing the 3 levels of resistance for lambda-cyhalothrin defined in Table 1 and zeta-cypermethrin.

Linear correlation of the LC_50_ values of lambda-cyhalothrin with type II pyrethroids alpha-cypermethrin, beta-cyfluthrin, and zeta-cypermethrin all produced slopes significantly different from zero (*P* < 0.05) with strongly supported relationships (*r*^2 ^= 0.58, 0.92, and 0.35, respectively, Fig. 3A–D). The linear correlation between lambda-cyhalothrin and zeta-cypermethrin included data from this study and from Rodbell and Wanner (2021) and Rodbell et al. (2022). In comparison, the correlation between lambda-cyhalothrin and type I pyrethroid bifenthrin failed to produce a slope significantly different from zero (*P* = 0.77, *r*^2^ = 0.0002, Fig. 3E). The correlation between lambda-cyhalothrin and permethrin was the most variable, and while the slope was significantly different from zero (*P* < 0.05), the relationship was the weakest (*r*^2^ = 0.28, Fig. 3F). This analysis also included data from Rodbell and Wanner (2021) and Rodbel et al. (2022). Several of the twelve sites produced contradictory relationships, indicating partial multiple resistance between permethrin and lambda-cyhalothrin (Vassiliou et al. 2010).

**Fig. 4.**
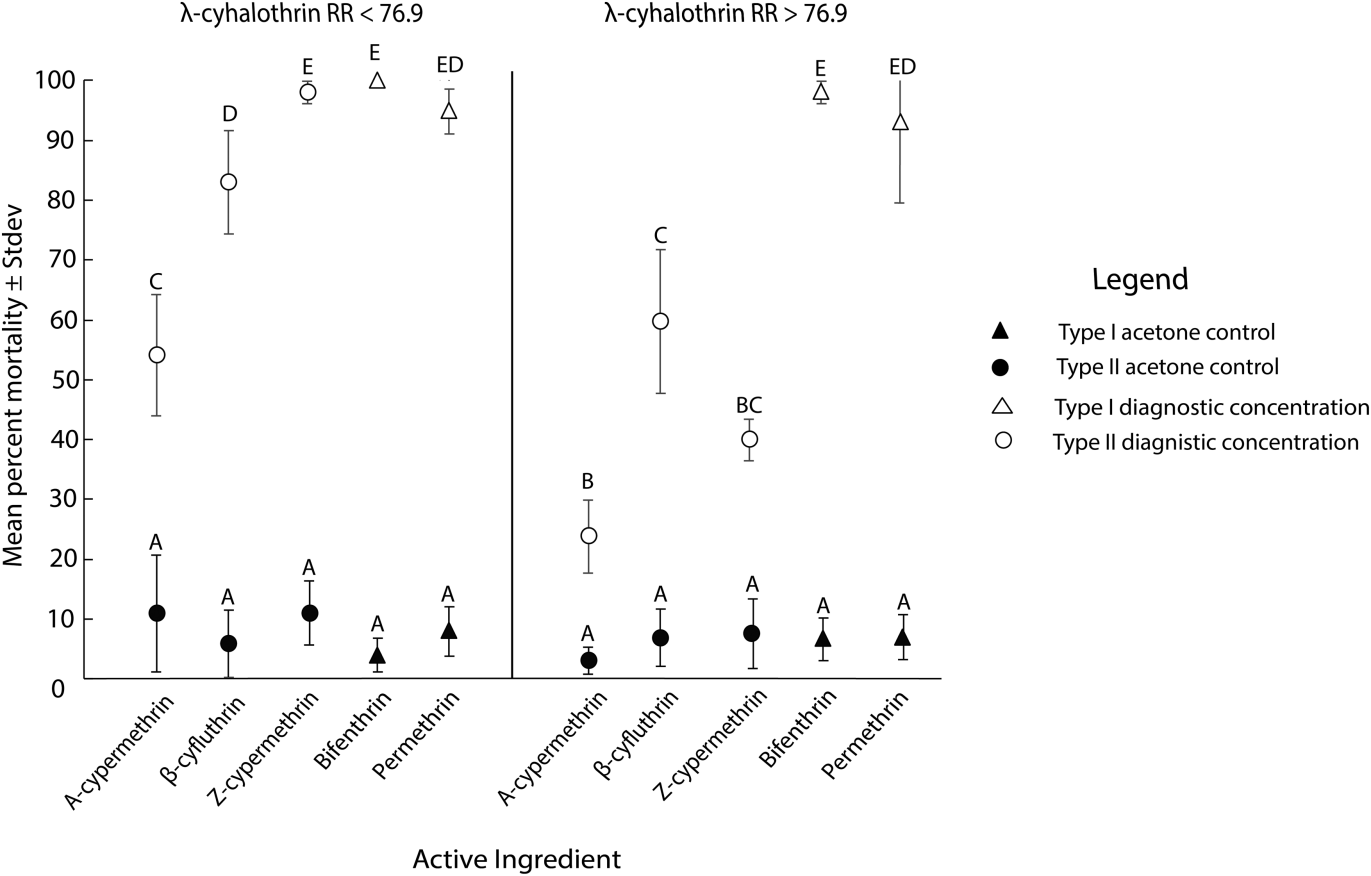
Mean percentage alfalfa weevil larval mortality with exposure to the diagnostic concentration of 3.3 µg/cm^2^ and the acetone control for all active ingredients tested, regardless of pyrethroid type, among location samples organized based on their resistance ratio (RR) to the type II pyrethroid, lambda-cyhalothrin (RR < 76.9 and RR > 76.9). In this instance, significant differences between treatments (*P <* 0.05) are denoted by different letters (*F *= 98; df = 20, 192; *P *< 0.001).

As a post hoc analysis, percentage mortality at a diagnostic concentration was further analyzed for multiple resistance among pyrethroid active ingredients. Larvae exposed to the acetone control never exceeded 20% mortality, averaging 3–11% (Fig. 4). At the diagnostic concentration, average mortality of larvae within the susceptible/moderate resistance lambda-cyhalothrin category (RR < 76.9) was between 83 and 100% for both type I and II active ingredients (Fig. 4), apart from alpha-cypermethrin (54%). Within the highly resistant lambda-cyhalothrin category (RR > 76.9) the pattern of mortality for type I and II active ingredients differed. Percentage mortality for type I pyrethroids bifenthrin and permethrin was 98 and 93% respectively, while type II pyrethroids produced significantly lower average mortalities, 23–60% (Fig. 4) (*F* = 98; df = 20, 192; *P *< 0.001). While permethrin produced evidence of intermediate multiple resistance with lambda-cyhalothrin (with a weak *r*^2^ value), resistance to permethrin can be overcome at higher concentrations (Fig. 4). Beta-cyfluthrin produced the strongest correlation with lambda-cyhalothrin (Fig. 3B), and low mortalities at the high discriminating concentration, suggesting it may be the least effective against resistant alfalfa weevils. Collectively, the laboratory bioassays support multiple resistance between type II pyrethroids and no or limited multiple resistance between pyrethroid types.

### Field Trials Support Multiple Resistance Between Type II Pyrethroids Across the Western US

Commercial insecticides with type I and II pyrethroid active ingredients were tested in commercial forage alfalfa fields located in AZ, MT, and WA, against alfalfa weevils highly resistant to lambda-cyhalothrin based on bioassay LC_50_ values (Table 1). In all 3 locations, type II pyrethroid insecticides failed to provide adequate control of the lambda-cyhalothrin-resistant alfalfa weevil larvae. Consistent with the bioassay results, bifenthrin and indoxacarb (MoA 22A) were effective. Differences in the average numbers of larvae 6–7 days after treatment were statistically significant (*P* < 0.05) at all 3 field trial sites. Generally, there were no significant differences between all type II pyrethroids and the type I pyrethroid permethrin. Brigade (AI., bifenthrin) was the only pyrethroid formulation that provided significant reductions in larval numbers compared to the type II pyrethroids and the control group (Figs. 1A–C, Table 4). Steward EC (AI., indoxacarb, MoA 22A) was included as an alternate MoA comparison. Regardless of location, Steward EC reduced alfalfa weevil populations compared to the control group, all type II pyrethroids, and permethrin (*P* < 0.001, Figs. 1A–C, Table 2) but was not significantly different from Brigade. The Big Horn County MT site had the highest alfalfa weevil population with the control plots yielding 1,300 larvae per 10 sweeps. While type II pyrethroids reduced these numbers by approximately 70%, the total numbers remained above the economic threshold of 20/sweep, unlike the results obtained from Brigade and Steward EC (Evans 1989, Blodgett 1996; Fig. 1B).

### Alfalfa Weevil Strain Identification

The specific alfalfa weevil strain(s) infesting each field site was determined using protocols developed by Erney et al. (1996). Larvae from the MT and WA field sites all produced 2 diagnostic bands (486 and 357 bp) for the western strain after digesting a COI/COII PCR product with Alu I. Larvae from the AZ field site all produced 3 diagnostic bands (357, 284, 202 bp), for the Egyptian/eastern strain. These larvae were all identified as Egyptian strain based on a 2 nucleotide polymorphism within a region of the Cytochrome b/ ND1 mitochondrial gene region, TT for the Egyptian strain while the eastern strain is AC (Fig. 2).

We note an error in the protocol of Erney et al. (1996), an error also noted by Kuwata et al. (2005). Erney et al. (1996) erroneously reported the use of reverse primer C2-N-3686 that produces a 927 bp PCR product rather than the reported 618 bp band. The result is an additional 309 bp of sequence that contains 1 additional AluI restriction site resulting in an additional 84 bp band common to all weevil strains (Supplementary File S1). Despite this discrepancy, the protocol accurately diagnoses the 3 different strains of alfalfa weevil (Kuwata et al. 2005).

## Discussion

Laboratory bioassays and a field trial in 2020 identified resistance to lambda-cyhalothrin in 2 commercial alfalfa fields in Big Horn County Montana (Rodbell and Wanner 2021). Resistance to lambda-cyhalothrin is now known to be established in the western United States (Rodbell et al. 2022). This study has demonstrated that all type II pyrethroids will not be effective at controlling lambda-cyhalothrin resistant populations, a pattern occurring for both western and Egyptian alfalfa weevils. However, alfalfa weevils in many areas remain susceptible, even when located in the same county where highly resistant sites were identified (Rodbell et al. 2022). With further research, specific IRM recommendations can be developed to mitigate the further development of resistance.

Cross- and multiple resistance among active ingredients within the same MoA group is common for insect pests (Yu 2015). Regardless of alfalfa weevil strain, laboratory bioassays illustrated a pattern of multiple resistance among type II pyrethroids and no, or limited, resistance between pyrethroid types, a consistent pattern in the 6 western states tested. Often, laboratory bioassays are not corroborated with field trial data because it is laborious, and applying bioassay results to field efficacy can be challenging, especially where there is a large range of LC_50_ values (Ball 1981, Denholm et al. 1984, ffrench-Constant and Roush 1990). Our laboratory results from 6 states were consistent with the 3 field trials conducted in distant and distinct alfalfa production systems, where lambda-cyhalothrin resistant alfalfa weevils were not controlled by type II pyrethroids (33–80% control). The type I pyrethroid, bifenthrin (registered for seed alfalfa but not forage alfalfa production), was the only pyrethroid effective against lambda-cyhalothrin resistant alfalfa weevils in lab and field trials (98–100% control in field trials), while type I pyrethroid, permethrin, produced mixed results. Collectively, this study demonstrates that all type II pyrethroid active ingredients and their commercial formulations will be ineffective at controlling alfalfa weevil in areas of the western United States where significant pyrethroid resistance has developed.

Similar degrees of resistance between type II pyrethroids were noted, the LC_50_ values of lambda-cyhalothrin were strongly correlated with the LC_50_ values of all other type II active ingredients tested, using data from multiple western region states (R^2^ 0.35–0.92, *P* < 0.05, Fig. 3) (Devries and Georghiou 1981, Scott 1990, Carriére et al. 1996, Vassiliou et al. 2010, Kostromytska et al. 2017, Menger et al. 2020). In contrast, larvae resistant to lambda-cyhalothrin were not mutually resistant to bifenthrin (LC_50_ values ranged from 0.01 to 0.86 µg/cm^2^, Fig. 3), the slope of the relationship was not significantly different from zero. This lack of mutual resistance did not extend to type I pyrethroid, permethrin, as LC_50_ values ranged from 0.47 to 100.00 µg/cm^2^, although field site variability of permethrin relative to lambda-cyhalothrin resulted in the weakest correlation (Fig. 3). This variability may reflect differences in historical use, since unlike bifenthrin, permethrin is registered for forage alfalfa production (Vardiman et al. 2022). As one of the original pyrethroids used in agriculture, permethrin has less persistence and is relatively less toxic compared to type II pyrethroids. Despite this difference, at a high discriminating concentration, permethrin was more toxic to lambda-cyhalothrin resistant larvae compared to the type II active ingredients, suggesting a degree of partial resistance. The range of LC_50_ values observed for bifenthrin may be the result of differences in size, the sex of the insect, low levels of cross-resistance or multiple resistance or migration from seed alfalfa production fields where bifenthrin is registered (Koehler et al 1993, Glunt et al. 2011, Zhu et al. 2020).

Similar patterns of multiple resistance among type II pyrethroids and moderate degrees of resistance to permethrin have occurred for other insect species and may indicate mechanism(s) of resistance. For example, *Anopheles funestus* in Cameroon were identified as resistant to the type II pyrethroids lambda-cyhalothrin and deltamethrin as well as the type I pyrethroid, permethrin. However, the degree of resistance was higher among the type II pyrethroids compared to permethrin (Menze et al. 2016). Greater resistance to type II pyrethroids compared to type I is consistent with a *super-kdr* target-site mutation. *Super-kdr* in other insects is sensitive to chemical structure, with a higher degree of resistance to type II pyrethroids (e.g., lambda-cyhalothrin) than type I pyrethroids (e.g., bifenthrin) (Khambay et al. 1994, Davies et al. 2007). In comparison, the *kdr* mutation has the same degree of resistance to all MoA 3A active ingredients, a pattern not observed in our study (Khambay et al. 1994, Davies et al. 2007). Whether the *super-kdr* mutation contributes to resistance of western United States alfalfa weevils remains to be determined. Alternatively, multiple mechanisms of resistance can occur simultaneously, resulting in the enhanced efficacy of each resistance mechanism, and produce distinct patterns of resistance to multiple active ingredients (Yu 2015).

In addition to target-site insensitivity, pyrethroid resistance can include behavioral avoidance, enhanced detoxification, and reduced cuticular penetration, alone or in combinations (Soderlund and Bloomquist 1990, Roberts et al. 1997, Yu 2015, Balabanidou et al. 2018, Haddi et al. 2018). For example, pyrethroid resistance in rice weevil (*Sitophilus oryzae*) strains is the result of target-site insensitivity (*kdr* mutation), behavioral avoidance, and enhanced detoxification (Haddi et al. 2018). Additionally, different combinations of resistance mechanisms were identified in rice weevil populations from Latin America compared to Australia and Turkey (Haddi et al. 2018). While we observed a consistent pattern of resistance, it remains possible that the mechanism(s) of pyrethroid resistance may vary in alfalfa weevil populations across the western United States.

Broad ranges of LC_50_ values were observed for all pyrethroid active ingredients included in this study (Tables 3 and 4). Such broad LC_50_ values have been reported in numerous studies that evaluated the susceptibility of field populations of insects (Luttrell et al. 1999, Shelton et al. 2003, Snodgrass and Scott 2003, Wang et al. 2010, Kostromytska et al. 2017, Rodbell and Wanner 2021, Rodbell et al. 2022). These broad ranges of LC_50_ values can be influenced by inherent variation in insect susceptibility, the intensity of resistance and technical variables of the bioassay (e.g., size, life stage, sex, and vigor of the field-collected insects) (Koehler et al. 1993, Glunt et al. 2011, Zhu et al. 2020).

Alfalfa is grown in 48 states and the production systems and climate conditions vary significantly. In the western US, annual harvests can range from 1 (e.g., dryland production) to as high as 10 (e.g., irrigated low desert production) where the forage is either used on-farm or sold as a commodity. Irrigation varies from none (dryland), to center-pivot sprinklers, to flood irrigation. Climate diversity influences alfalfa weevil phenology (Stilwell et al. 2010). For example, oviposition to new adult eclosion requires less than 3 mo in cold semi-arid and hot desert climates (e.g., Köppen climate types), where cold dormancy can last 6 mo, and in hot climates, estivation can last 8 mo. These contrast warm-summer Mediterranean climates where temperate conditions allow prolonged larval and adult activity. Diversity of climate and alfalfa production influence IPM and IRM strategies that need to be adapted for local abiotic and biotic conditions. For example, the alfalfa weevil degree-day model that predicts development varies in different climates and it has been adapted to specific alfalfa weevil strains (Stilwell et al. 2010, UC-IPM 2014, Pellissier et al. 2017, USPEST 2023). Similarly, economic thresholds are affected by irrigation status, stand health, and yield as well as end-use of the alfalfa (Harrington et al. 2021).

The general principles of IRM have been established for more than 5 decades; their aim is to reduce selective pressure and slow the development of insecticide resistance traits. The most effective strategy is consistent with the principles of IPM, using methods such as cultural and biological control to reduce the frequency of insecticide applications that are applied only when economically necessary. Optimizing insecticide efficacy (e.g., calibrating equipment, timing applications, adjuvants, and using appropriate rates) helps delay resistance by overcoming insect defenses and increasing mortality of individuals heterozygous for resistance. When insecticides are used more frequently, the most common strategy to delay resistance is rotating their MoA to reduce selection on any 1 MoA group or sub-group, a challenge for the alfalfa weevil where MoAs are limited in forage alfalfa. One continuing challenge of IRM is to provide producers with recommendations tailored to the specific pest and its cropping system.

The time required for insecticide susceptibility to reestablish in specific pest populations is generally unknown and is related to factors such as the fitness cost associated with resistant genotypes and the availability of susceptible populations for mating. Our study did not test this objective with controlled experiments, but bioassay and field data collected over 4 consecutive years from the same ranch provides an estimate. Big Horn County Field Site 1 (Table 3) was highly resistant to lambda-cyhalothrin in 2020 (Rodbell and Wanner 2021, Rodbell et al. 2022). After 4 yr without pyrethroid use (indoxacarb was applied to control the outbreak levels of alfalfa weevil), larval control by lambda-cyhalothrin (applied in small research plots) increased from 0 to 60–80% and LC_50_ values decreased from > 3.33 µg/cm^2^ (failed to cause 50% mortality at the highest dose tested) to 0.34 µg/cm^2^ (Supplementary File S2 A–C). The degree of resistance to lambda-cyhalothrin declined from highly to moderately resistant.

In areas of high pyrethroid resistance defined in Rodbell et al (2022): Indoxacarb (MoA 22A), that maintains its effectiveness regionally, can reduce outbreak populations to endemic levels when applied for several consecutive years. During this period, resistance levels may decrease sufficiently to allow pyrethroids (MoA 3A) back into rotational use. Registering new MoA groups for alfalfa weevil control in forage alfalfa is a priority.

For susceptible and moderately resistant areas defined in Rodbell et al (2022): We recommend using pyrethroids (MoA 3A) and indoxacarb (MoA 22A) no more than once every 3 yr. IPM strategies such as early harvest need to be integrated with chemical control tactics to limit the overuse of registered chemicals for the control of this pest. However, additional IPM options need to be developed. For example, early harvest does not fit into all production systems since larval development may not be uniform and pest pressure can carry over to the next cutting, risking continued damage to the stand (Blodgett 1996, Cornell 2023). Furthermore, economic thresholds, a critical component of IPM, need to be updated and customized to different production systems and climates of the western region (Harrington et al. 2021). As new MoAs and IPM options become available the rotation strategy should be expanded. In the interim, older organophosphate insecticides such as dimethoate (MoA 1B) appear to be effective in some, but not all regions, alone or mixed with pyrethroids to improve control of resistant individuals (Wanner, unpublished data). Regional trials should be conducted to establish timing and efficacy of older chemistries that may have their own resistance concerns, as well as identifying the most susceptible life stage of the alfalfa weevil for these control options.

Monitoring to detect resistance and early intervention is a critical principle of IRM, but resistance is usually first discovered at control failure in the field. Based on our results, a discriminating bioassay (24 h contact assays using glass vials) will reliably detect pyrethroid resistance prior to field failure. We found exposing larvae to heat to be a more reliable method of determining mortality, as the larvae tended to curl and often did not respond to probing. For type I pyrethroids, vials should be treated with 1 ml of a solution at a concentration of 189.0 µg/ml, for type II pyrethroids vials should be treated with a concentration of 18.9 µg/ml, and for indoxacarb, due to its high efficacy, 2 concentrations are encouraged, 27 µg/ml and 8.2 µg/ml. It will also be important to monitor for early signs of resistance to indoxacarb as its use increases and to standardize alfalfa weevil bioassays for indoxacarb resistance. Future genomic research should focus on identifying molecular markers and the associated mechanisms of resistance that can be used to design higher throughput, more sensitive, and lower cost resistance monitoring assays.

Similar degrees of resistance among pyrethroid active ingredients in alfalfa weevil populations illustrates an emerging agricultural challenge in the western United States. Future research efforts are needed to determine other alfalfa weevil populations within North America that may follow similar patterns of multiple resistance. IRM recommendations presented in this study should be revisited as further research becomes available. Finally, Extension efforts are needed to deliver IRM and IPM tactics customized to local productions systems, to ensure sustainable alfalfa weevil management while limiting the use of chemical controls.

## Supplementary Material

toad218_suppl_Supplementary_File_S1

toad218_suppl_Supplementary_File_S2

toad218_suppl_Supplementary_Table_S1

toad218_suppl_Supplementary_Method_S1
