# Supplementary material for: Alfalfa weevils (Coleoptera: Curculionidae) in the western United States are resistant to multiple type II pyrethroid insecticides
Source: J Econ Entomol. 2023 Nov 25;117(1):280–92. doi: 10.1093/jee/toad218 (PMC10860156; doi:10.1093/jee/toad218)
Supplement: toad218_suppl_Supplementary_File_S1 [file toad218_suppl_supplementary_file_s1.docx]

**Supplementary File S1.**

The protocol described by Erney et al. (1996) was validated using alfalfa weevil samples from an alfalfa field near the University of California, Davis CA. Suppl. Fig. 1a illustrates the 927 base pair (bp) PCR product generated by primers C1-J-2797 and C2-N 3686 reported in Erney et al. (1996) and the three bands (486, 357 and 84 bp) that result from its digest with the AluI restriction enzyme, when the source is the western strain of alfalfa weevil (first larva analyzed from CA, Suppl. Fig. 1a). When eastern or Egyptian strain weevils are used as the source for PCR, digest with AluI produces four bands of different sizes (357, 284, 202 and 84 bp) (second larva analyzed from CA, Suppl. Fig. 1b). To identify the second larva as Egyptian or eastern strain primers CB-J-11545 and N1-N-11841 were used to amplify and sequence a 300 bp region of the mitochondrial Cytochrome b and ND1 genes. This segment of mitochondrial DNA contains two nucleotides that diagnose each strain (Suppl. Fig. 1c, eastern = AC; Egyptian = TT; and western = TC) (Erney et al. 1996). The nucleotide sequence from the second CA larva was TT, diagnosing it as the Egyptian strain. Using this protocol we identified a mixture of Egyptian and western strains from a single alfalfa field in CA.

Our results have one discrepancy compared to the protocol described by Erney et al. (1996). PCR primers C1-J-2797 and C2-N-3686 produced a 927 bp band compared to the 618 bp band reported by Erney et al. (1996). To resolve this discrepancy we sequenced our 927 bp PCR product obtained from the California western and Egyptian strain weevils. After analyzing the sequences we came to the conclusion that Erney et al. (1996) erroneously reported the use of reverse primer C2-N-3686, and rather used a different reverse primer inside the 927 bp region amplified by primers C1-J-2797 and C2-N-3686. The result is an additional 309 bp of sequence that contains one additional AluI restriction site in all weevil strains. Therefore, our digest with AluI produces one extra band. A study of weevil strains introduced to Japan also followed the protocol of Erney et al. (1996) and used the same C1-J-2797 and C2-N-3686 primers. Those authors came to the same conclusion, that Erney et al. (1996) erroneously reported the use of reverse primer C2-N-3686 (Kuwata et al. 2005). This Japanese study obtained results identical to ours reported in Suppl. Fig. 1. Despite this discrepancy the protocol accurately diagnoses the three different strains of alfalfa weevil (Kuwata et al. 2005).


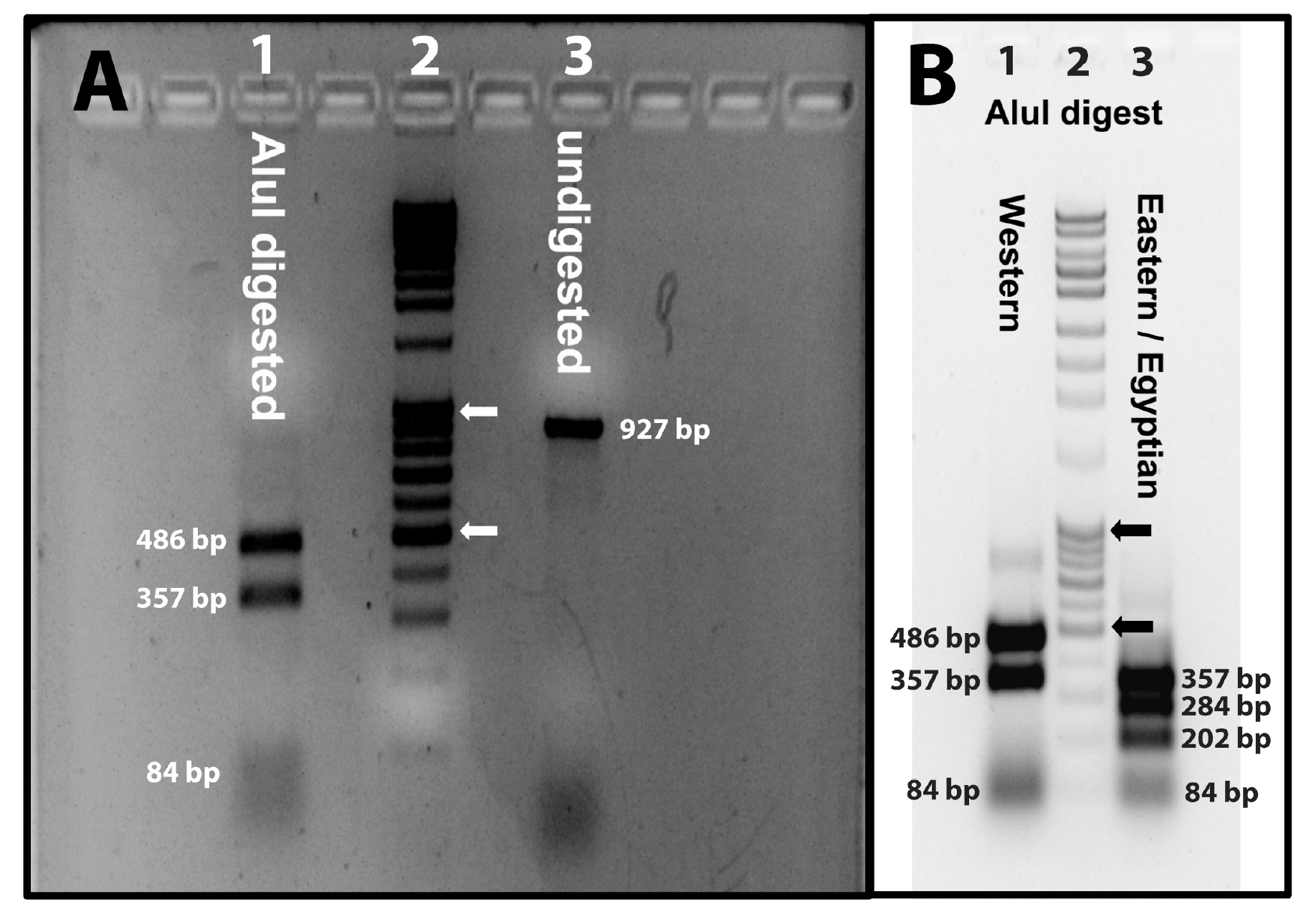


**C**

**
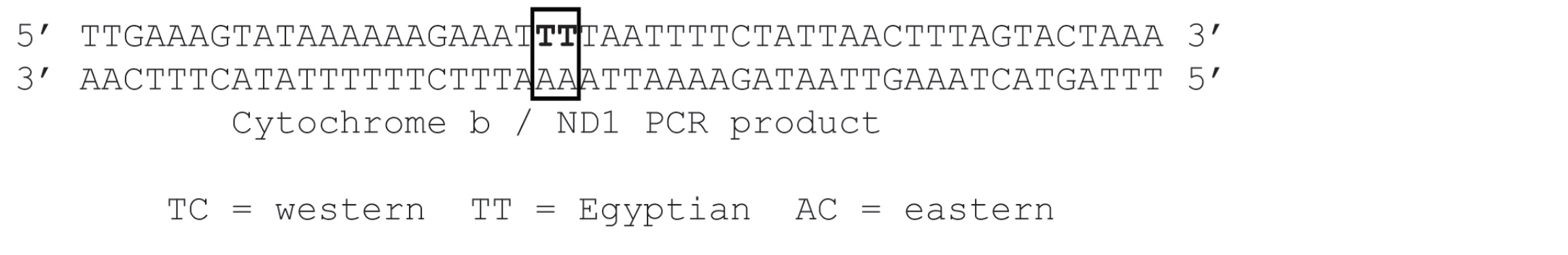
**

**Supplementary File 1.** Molecular genetic identification of eastern, Egyptian and western alfalfa weevils. **A)** First CA larval sample (western strain). PCR with C1-J-2797 and C2-N-3686 primers produces a 927 bp product (lane 3). When the western strain is the source of the band, its digestion with AluI restriction enzyme produces three smaller bands sized 486, 357 and 84 (lane 1). **B)** First (lane 1) and second (lane 2) CA larval samples. When the eastern or Egyptian strains are used, digestion of the PCR product with AluI restriction enzyme produces a different banding pattern, four bands sized 357, 284, 202 and 84 bp (lane 3) compared to the western strain (lane 1). Lane 2, 1 Kb plus DNA ladder, arrows indicate the 1000 and 500 bp markers. The 1.2% agarose gel in Fig. B provides better resolution of the small 84 bp band compared to the 0.8% agarose gel. **C)** Two nucleotides within a region of the Cytochrome b / ND1 mitochondrial gene region can diagnose the three strains of alfalfa weevil. TC = western strain; TT = Egyptian strain; and AC = eastern strain. Sequence from the second CA lava diagnosed it as the Egyptian strain.

**References Cited**

Erney SJ, Pruess KP, Danielson SD, Powers TO. Molecular differentiation of alfalfa weevil strains (Coleoptera: Curculionidae). Ann. Entomol. Soc. 1996:89:804-811.

Kuwata R, Tokuda M, Yamaguchi D, Yukawa J. Coexistence of two mitochondrial DNA haplotypes in Japanese populations of *Hypera postica* (Col., Curculionidae). J. Appl. Entomol. 2005:129:191-197.
