# Supplementary material for: Alfalfa weevils (Coleoptera: Curculionidae) in the western United States are resistant to multiple type II pyrethroid insecticides
Source: J Econ Entomol. 2023 Nov 25;117(1):280–92. doi: 10.1093/jee/toad218 (PMC10860156; doi:10.1093/jee/toad218)
Supplement: toad218_suppl_Supplementary_File_S2 [file toad218_suppl_supplementary_file_s2.docx]

| **Year** | **LC_50_ µg/cm^2^** | **T-ratio _slope_** | **χ^2^_df_** | ***P* value** | **RR** | **R Level** |
| --- | --- | --- | --- | --- | --- | --- |
| **2020** | >3.3 | 4.12 | 9.78_5_ | 0.08 | >256 | High |
| **2021** | 4.50 | 6.48 | 4.63_4_ | 0.33 | 346 | High |
| **2022** | 7.40 | 3.09 | 10.31_5_ | 0.07 | 569.23 | High |
| **2023** | 0.34 | 9.89 | 4.62_5_ | 0.46 | 26.15 | Moderate |

**B.**

**C.**

**Supplemental File S2.** A) Lambda-cyhalothrin (type II pyrethroid MoA 3A) LC_50_ values (µg/cm^2^) via laboratory bioassays conducted with location samples collected in 2020 and in 2023 collected from the same field site in Big Horn County MT (Big Horn County Montana Field Site 1). Big Horn County Montana Field Site 1 last applied pyrethroids in 2019. Resistance ratios (RR) and probit analysis statistics are presented for the *t-*ratio of the slope, chi-square (χ^2^), and the degrees of freedom (df). The degree of a location samples resistance to lambda-cyhalothrin was based on the LC_50_ value and its associated resistance ratio (Table 1). Three resistance categories are represented: susceptible (S); moderate (M); and high (H). Mean percentage mortality at a concentration at 3.3µg/cm^2^ of lambda-cyhalothrin illustrates a clear trend of improved efficacy over the four-year period of repeated laboratory bioassays. After four-years of exclusive use of Steward EC^®^, Warrior II^®^ was able to significantly reduce alfalfa weevil resistance to lambda-cyhalothrin, reducing the degree of resistance from high to moderate. A pattern corroborated by field trial data (C).
