# Supplementary material for: Alfalfa weevils (Coleoptera: Curculionidae) in the western United States are resistant to multiple type II pyrethroid insecticides
Source: J Econ Entomol. 2023 Nov 25;117(1):280–92. doi: 10.1093/jee/toad218 (PMC10860156; doi:10.1093/jee/toad218)
Supplement: toad218_suppl_Supplementary_Table_S1 [file toad218_suppl_supplementary_table_s1.docx]

**Supplemental Table S1.** Active ingredient by state, county, and field site that had one outlier concentration removed from the dataset. The table lists the concentration that was removed.

| **Active Ingredient** | **Year** | **State** | **County** | **Field Site** | **Concentration # Removed** |
| --- | --- | --- | --- | --- | --- |
| Alpha-cypermethrin | 2021 | MT | Big Horn | 1 | 1 |
| Lambda-cyhalothrin | 2022 | MT | Big Horn | 1 | 1 |
|  |  |  | Madison | 1 | 5 |
|  |  | OR | Umatilla | 1 | 5 |
|  |  | WA | Yakima | 1 | 4 |
| Zeta-cypermethrin | 2021 | MT | Madison | 1 | 2 |
| Bifenthrin | 2022 | MT | Big Horn | 1 | 4 |
|  |  |  |  | 2 | 1 |
|  |  | WA | Klickitat | 2 | 2 |
| Permethrin | 2022 | OR | Umatilla | 1 | 4 |
|  |  | WA | Klickitat | 1 | 1 |
|  |  |  | Yakima | 1 | 2 |
|  |  | WY | Sheridan | 1 | 4 |
| Indoxacarb- MoA 22A | 2022 | OR | Umatilla | 1 | 3 |
