# Supplementary material for: Alfalfa weevils (Coleoptera: Curculionidae) in the western United States are resistant to multiple type II pyrethroid insecticides
Source: J Econ Entomol. 2023 Nov 25;117(1):280–92. doi: 10.1093/jee/toad218 (PMC10860156; doi:10.1093/jee/toad218)
Supplement: toad218_suppl_Supplementary_Method_S1 [file toad218_suppl_supplementary_method_s1.docx]

**Supplementary Methods S1**

Glass vials (Discount Vials, Madison, WI) were treated with 1 mL of an insecticide concentration and diluent 95% acetone was evaporated without heat on a commercial food roller (WebstaurantStore, Lititz, PA) at room temperature (21°C) (Brindley 1975). Resulting in insecticide concentrations of 0.0033-10.0 µg/cm^2^ for type II pyrethroids (e.g., lambda-cyhalothrin), 0.033-100.0 µg/cm^2^ for type I pyrethroids (e.g., bifenthrin), and 0.0001-0.1 µg/cm^2^ for indoxacarb (MoA 22A). Once treated, glass vials were stored in a refrigerator (4°C) and used within a two-week period. Each bioassay typically included seven concentrations and a 95% acetone control (n = 5 replicated vials, 10 larvae per vial). After 24-hours in the dark at room temperature, the larvae were scored as dead or alive after exposure on a heated hot plate (43-50 °C) (Rodbell and Wanner 2021, Rodbell et al. 2022).

Probit analysis was used to quantify the lethal concentration of an active ingredient that generated 50% mortality (LC_50_) using Probit Or LOgit analysis (POLO) software (LeOra Software, Parma, MO) (Robertson et al. 2017). Bioassays with control mortality >20%, t-ratio of the slope less than 1.96, and/or chi-square (χ^2^) goodness of fit test p < 0.05 were omitted (Robertson et al. 2017). If mortality was observed in the control group, correction for natural response was estimated by the POLO probit analysis (Robertson et al. 1980). Data that did not fit the probit model and χ^2^ goodness of fit test were omitted unless a single outlier was identified as the cause for the lack of fit. Outliers were identified through the graphical visualization of the residual values (Sarkar et al. 2011, Robertson et al. 2017). Single outliers were removed from one alpha-cypermethrin, four lambda-cyhalothrin, one zeta-cypermethrin, three bifenthrin, four permethrin, and two indoxacarb bioassays (Supp. Table S1). Larval mortality from some highly resistant sites failed to reach 50% and did not fit the requirements of probit analysis, these LC_50_ values was conservatively listed as greater than (>) the highest concentration tested (Brindley 1975, Haddi et al. 2018, Rodbell and Wanner 2021, Rodbell et al. 2022).

Probit analysis was used to quantify the lethal concentration of an active ingredient that generated 50% mortality (LC_50_) using Probit Or LOgit analysis (POLO) software (LeOra Software, Parma, MO) (Robertson et al. 2017). Bioassays with control mortality >20%, t-ratio of the slope less than 1.96, and/or chi-square (χ^2^) goodness of fit test p < 0.05 were omitted (Robertson et al. 2017). If mortality was observed in the control group, correction for natural response was estimated by the POLO probit analysis (Robertson et al. 1980). Data that did not fit the probit model and χ^2^ goodness of fit test were omitted unless a single outlier was identified as the cause for the lack of fit. Outliers were identified through the graphical visualization of the residual values (Sarkar et al. 2011, Robertson et al. 2017). Single outliers were removed from one alpha-cypermethrin, four lambda-cyhalothrin, one zeta-cypermethrin, three bifenthrin, four permethrin, and two indoxacarb bioassays (Supp. Table S1). Larval mortality from some highly resistant sites failed to reach 50% and did not fit the requirements of probit analysis, these LC_50_ values was conservatively listed as greater than (>) the highest concentration tested (Brindley 1975, Haddi et al. 2018, Rodbell and Wanner 2021, Rodbell et al. 2022).

**References Cited**

Brindley WA. Insecticide bioassays with field location samples of alfalfa weevils, a simplified approach. J. Econ. Entomol. 1975:68:227-230.

Haddi K, Valbon WR, Jumbo LOV, de Oliveira LO, Guedes RNC, Oliveira EE. Diversity and convergence of mechanisms involved in pyrethroid resistance in the stored grain weevils, *Sitophilus* spp. Sci. Rep. 2018:8:1-15.

Robertson JL, Jones MM, Olguin E, Alberts B. Bioassays with Arthropods. 3^rd^ ed. Boca Raton (FL): CRC Press; 2017.

Robertson J, Russell R, Savin NE. 1980. POLO: a user’s guide to Probit Or Logit analysis. Berkley (CA): United States Department of Agriculture Forest Service; 1980. [accessed 2023 June 1]. https://www.fs.usda.gov/psw/publications/documents/psw_gtr038/psw_gtr038.pdf

Rodbell EA, Hendrick ML, Grettenberger IM, Wanner KW. Alfalfa weevil (Coleoptera: Curculionidae) resistance to lambda-cyhalothrin in the western United States. J. Econ. Entomol. 2022:115:2029-2040.

Rodbell EA, Wanner KW. First report of alfalfa weevil (Coleoptera: Curculionidae) resistance to lambda-cyhalothrin in Montana. J. Econ. Entomol*.* 2021:114:2088-2095.

Sarkar SK, Midi H, Rana S. Detection of outliers and influential observations in binary logistic regression: an empirical study. J. Appl. Sci. 2011:11:26-35.
